# Effect of Deep Cryogenic Treatment on Cyclic Fatigue of Endodontic Rotary Nickel Titanium Instruments

**DOI:** 10.22037/iej.2017.42

**Published:** 2017

**Authors:** Mohammad Yazdizadeh, Masoumeh Skini, Seyyed Mohsen Hoseini Goosheh, Mansour Jafarzadeh, Milad Shamohammadi, Vahid Rakhshan

**Affiliations:** a *Department of Endodontics, Dental School, Ahvaz Jundishapur University of Medical Science, Ahvaz, Iran; *; b * Department of Orthodontics, Dental School, Ahvaz Jondishapoor University of Medical Science, Iran;*; c * Researcher, Tehran, Iran*

**Keywords:** Cryogenic Treatment, Cyclic Fatigue, Instrument Fracture, Rotary Nickel Titanium Files

## Abstract

**Introduction:**

Cyclic fatigue is the common reason for breakage of rotary instruments. This study was conducted to evaluate the effect of cryogenic treatment (CT) in improving the resistance to cyclic fatigue of endodontic rotary instruments.

**Methods and Materials:**

In this *in vitro* study, 20 RaCe and 20 Mtwo files were randomly divided into two groups of negative control and CT. CT files were stored in liquid nitrogen at -196^°^C for 24 h, and then were gradually warmed to the room temperature. All files were used (at torques and speeds recommended by their manufacturers) in a simulated canal with a 45^°^ curvature until breakage. The time to fail (TF) was recorded and used to calculate the number of cycle to fail (NCF). Groups were compared using independent-samples t-test.

**Results:**

Mean NCFs were 1248.2±68.1, 1281.6±78.6, 4126.0±179.2, and 4175.4±190.1 cycles, for the Mtwo-control, Mtwo-CT, RaCe-control, and RaCe-CT, respectively. The difference between the controls and their respective CT groups were not significant (*P*>0.3). The difference between the systems was significant.

**Conclusion:**

Deep CT did not improve resistance to cyclic fatigue of the evaluated rotary files.

## Introduction

Modern endodontics is greatly benefited from the advent of nickel-titanium (NiTi) rotary instruments which can bend while rotating even in severely curved canals, allowing a much faster and more convenient preparation of curved and flat canals with minimum transportation [1-6]. NiTi instruments possess extraordinary shape memory and pseudoelasticity, very high flexibility, low modulus of elasticity, great resistance to torsional fractures, wide range of elastic deformation and high overall strength [2, 5-9]. Despite these desirable properties, rotary NiTi files are vulnerable to breakage, due to their mode of action in continually rotational movement. In few cases, when file tip is stuck in apical area during instrument rotation, torsional fatigue leads to ductile fracture [1, 5, 10]. The more common cause of instrument separation is cyclic fatigue. When file is rotating within a curved canal, any given point on file undergoes alternating compression and tension, especially at points close to canal curvature . The continuous swapping between severe compressive and tensile stresses at each point of the bent instrument cause cyclic fatigue and result in departure coined brittle fracture [1, 2, 5, 7, 11], which happens without any warning, unlike in stainless steel files [1, 5]. This mechanism is responsible for up to 90% of fractures [1, 5, 10]. 

Numerous methods have been proposed to improve the resistance of rotary files to cyclic fatigue. These include surface treatments and cryogenic treatment (CT) [12]. Historically, surface hardness and thermal stability of metals were improved by cooling to temperatures around -60 to -80^°^C [2, 7, 13]. For the past four decades, a new modification of the conventional cold treatment has been shown promising in improvement of metal properties such as overall strength with efficacies much better than conventional cold treatment. This technique uses super cooled bathing at -196^°^C (deep CT) or storage in proximity to such super cooled materials (dry or shallow CT) [1, 2, 5-7, 11, 13-15]. Unlike surface treatments which exclusively modify alloy surface, this method alters the entire material mass by improving the transformation extent of the martensitic phase, the precipitation of the eta particles, and the release of internal stresses of alloy as a result of CT-induced plastic transformation [2, 7, 11, 14-16]. Therefore, it might be useful for strengthening rotary endodontic files [7].

Despite the importance of this subject, studies assessing any effects of cryogenic treatment on NiTi files are scarce [7]. Studies on effect of CT on cyclic fatigue resistance are even less common. One study examined the effect of deep CT on a new martensitic NiTi alloy which is not as commonly used as other NiTi instruments [1]. The other one evaluated effects of dry/shallow CT, and not effects of deep CT method [5]. This *in vitro* study have assessed the effect of deep CT on cyclic fatigue resistance of commonly used NiTi files. Its null hypotheses were lack of differences between cyclic fatigue of two rotary systems and lack of differences between cyclic fatigue of files treated or not treated with deep CT.

## Materials and Methods

This *in vitro* study was performed on 40 rotary NiTi files divided into 2 groups of RaCe (FKG Dentaire, La Chaux-de-Fonds, Switzerland) and Mtwo (VDW GmbH, Munich, Germany) (*n*=20), each with two subgroups of negative control and cryogenic treatment (*n*=10). Tip sizes of all files were 25/0.06.

Specimens in CT subgroups were stored in liquid nitrogen at -196^°^ C for 24 h. Afterwards, they were removed from nitrogen container and were placed at room temperature (about 25^°^C) to gradually warm up to room temperature [1, 7].

***Cyclic fatigue resistance***

A device was made to standardize the rotation of files. The device consisted of a stainless steel cast block, with a simulated canal formed within it. The simulated canal was a negative template of a 0.06-tapered gutta-percha with a tip size of 25 and a 45^°^ curvature. Each endodontic file would be rotated within this canal. For this purpose, the file was attached to a low-speed handpiece (ER64, NSK, Tokyo, Japan) and they were placed in proximity to the metal block (using a plastic retainer holding the handpiece) in a way that the file was completely inserted into the canal up to the apex. The handpiece was attached to a rotary motor with (DW11506-00-30, Aseptico, Woodinville, WA, USA). A tempered glass was used to cover the block and its canal in order to stabilize the instrument while also providing direct visualization.

The retainer allowed adjustment of the handpiece in order to accommodate the instrument up to the apex, at a relaxed position. The torque-controlled motor was used to rotate the instruments according to their manufacturers’ instructions. RaCe files were rotated 800 rpm at a 150-g/cm torque. Mtwo files were rotated 280 rpm at a 230-g/cm torque. The canal was lubricated using 2.5% sodium hypochlorite. Each instrument was rotated until breakage, under the direct supervision of an endodontist. A digital stopwatch was used to measure the rotation time. The time to fail (TF) was used to calculate the number of cycles to fail (NCF=rounds per second × time [12]), as a direct indicator of cyclic fatigue.

***Statistical analysis***

Descriptive statistics were calculated for NCF and TF. A Kolmogorov-Smirnov test showed that all groups were normally distributed. Two-way analysis of variance (ANOVA) was used to assess effect of cryogenic treatment and brands on NCF. Independent-samples t-test of SPSS program (SPSS, Chicago, IL, USA) was used to compare the control groups with their respective CT groups. It was also used to compare both file systems with each other. The level of significance was pre-determined as 0.05.

## Results

Results of this study pertaining to NCF and TF are presented in Table 1. ANOVA showed a significant difference between brands (*P*=0.000) but not between control group and cryogenic treatment (*P*=0.358). Interaction of these variables was not significant (*P*=0.858). Results pertaining to NCF indicated a much higher resistance of RaCe files to cyclic fatigue compared to Mtwo files. The difference between the systems was significant either for files undergone cryogenic treatment (difference=2893 cycles, *P*=0.000) or for the negative controls from both systems (difference=2887 cycles, *P*=0.000). However, the effect of cryogenic treatment on NCF was not significant, either for Mtwo (*P*=0.323) or for RaCe (*P*=0.557).

Both systems showed working times (TF) very close to each other. The difference between TF of two systems was small but still statistically significant either for files undergone cryogenic treatment (difference=53 sec, *P*=0.000) or for negative controls from both systems (difference=42 sec, *P*=0.000). However, effect of cryogenic treatment on TF was not significant, either for the Mtwo system (*P*=0.323) or for the RaCe system (*P*=0.557). 

## Discussion

Findings of this study showed that 24 h of deep CT cannot considerably improve the life time and fatigue resistance of evaluated NiTi rotary files. Results of the current study were contrasting those of George *et al.* [5], who showed 20% to 60% significant improvements in number of cycles to fail of 3 different rotary files after dry cryogenic treatment. Our results were also in contrast to findings of Vinothkumar *et al.* [1], showing a significant 10% increase in fatigue resistance as indicated by NCF [1]. The reason for the controversy might be methodological differences. For instance, canal curvature was 30 degrees in the aforementioned study, which would allow a more relaxed rotation of file and less severe compressive/tensile forces compared to that happened in 45^°^ curved canal of this study. Another reason for the difference between this study and that of Vinothkumar *et al.* [1] might be the used alloys. Vinothkumar *et al. *[1] used a rotary system made from a newly introduced martensitic NiTi alloy (HyFlex CM, Coltene Whaledent, Cuyahoga Falls, OH, USA) [1]. This alloy might have a greater portion of complete martensite phase compared to conventional NiTi alloys [1, 11, 17]. A more complete martensite transformation and an increase in the martensite-to-austenite ratio in the alloy might be one of the main mechanisms believed to be responsible for the effect of cryogenic treatment [2, 11, 14-16]. Therefore, perhaps the production protocol of these new martensitic rotary files has made them prone to more cryogenic alterations. This should be assessed in future studies. Another study showed that 24 h of deep cryogenic treatment increases the martensitic phase of these already-enhanced alloys without changing their grain sizes [11]. As a side note, manufacturer of these new files (HyFlex CM) claims that they might have about 300% greater resistances to cyclic fatigue compared to other rotary NiTi files. However, that TF claimed by the manufacturer (183 sec) was smaller than that of both conventional NiTi systems that were tested in this study. In addition, it was showed that without CT, an average of 2011 cycles would be needed to break the file, within 241 sec of rotation at 500 rpm (with an NCF of 2008 cycles). This TF was again smaller than the TF observed in this study and its corresponding NCF was comparable to results of Mtwo but much lower than NCF of RaCe observed in this study.

George *et al. *[5] used 45 degrees of curvature similar to this study and detected significant improvements as well; nonetheless their method of CT was dry type and differed from that of this study. In addition, George *et al. *[5] used the same rotation speed for all three tested systems, regardless of their manufacturers’ instructions. Perhaps this might be one of reasons for their very low NCFs, being all below about 270 to 450 cycles in the negative control and up to 710 cycles in the CT group; which these were much smaller than our findings, especially in the case of RaCe instruments which had about 4100 cycles to fail in this study.

Our findings were similar to those of George *et al.* [5] who showed different cyclic fatigue resistances for various systems. Each of these systems has its own chemical composition, cross-section, working protocol, *etc*. In this study, RaCe had cyclic fatigue resistances about twice as that of Mtwo. The very small coefficients of variation confirm these results as reliable. RaCe instruments have a triangular cross section and possess alternating cutting edges, which might reduce the working torque and prevent the file blockage [18]. However, Mtwo instruments have two cutting edges with sharp posterior aspects in order to increase their cutting efficacy, facilitate their movement, and increase their handling [18].

This study was limited by some factors. Rotation speeds and torques of both systems were not standardized. However, standardizing these factors was impossible, because these files cannot function at their optimum strength under circumstances other than those recommended by the manufacturers. Also clinicians would not use them at other parameters. Therefore, standardizing them like in some earlier results would render the results unreliable. This was the reason that although the NCF of the files differed greatly, their working times were very close to each other.

**Table 1:** Study results in different subgroups

			**Mean (SD)**
**Number of cycles to fail**	**Mtwo**	**None**	1248.2 (68.1)
		**Cryogenic**	1281.6 (78.6)
	**RaCe**	**None**	4126.0 (179.2)
		**Cryogenic**	4175.4 (190.1)
**Time to fail (sec)**	**Mtwo**	**None**	267.6 (14.6)
		**Cryogenic**	274.8 (16.9)
	**RaCe**	**None**	309.5 (13.4)
		**Cryogenic**	313.2 (14.3)

## Conclusion

It can be concluded that RaCe has a better resistance to cyclic fatigue compared to Mtwo. Deep cryogenic treatment for 24 h did not improve the cyclic fatigue resistance of these rotary instruments.
